# Soil microbial responses to nitrogen addition in arid ecosystems

**DOI:** 10.3389/fmicb.2015.00819

**Published:** 2015-08-14

**Authors:** Robert L. Sinsabaugh, Jayne Belnap, Jennifer Rudgers, Cheryl R. Kuske, Noelle Martinez, Darren Sandquist

**Affiliations:** ^1^Biology Department, University of New Mexico, AlbuquerqueNM, USA; ^2^Southwest Biological Science Center, U.S. Geological Survey, MoabUT, USA; ^3^Bioscience Division, Los Alamos National Laboratory, Los AlamosNM, USA; ^4^California State University, FullertonCA, USA

**Keywords:** arid ecosystems, nitrogen deposition, microbial biomass, ecoenzyme activity, meta-analysis

## Abstract

The N cycle of arid ecosystems is influenced by low soil organic matter, high soil pH, and extremes in water potential and temperature that lead to open canopies and development of biological soil crusts (biocrusts). We investigated the effects of N amendment on soil microbial dynamics in a *Larrea tridentata-Ambrosia dumosa* shrubland site in southern Nevada USA. Sites were fertilized with a NO_3_-NH_4_ mix at 0, 7, and 15 kg N ha^-1^ y^-1^ from March 2012 to March 2013. In March 2013, biocrust (0–0.5 cm) and bulk soils (0–10 cm) were collected beneath *Ambrosia* canopies and in the interspaces between plants. Biomass responses were assessed as bacterial and fungal SSU rRNA gene copy number and chlorophyll *a* concentration. Metabolic responses were measured by five ecoenzyme activities and rates of N transformation. By most measures, nutrient availability, microbial biomass, and process rates were greater in soils beneath the shrub canopy compared to the interspace between plants, and greater in the surface biocrust horizon compared to the deeper 10 cm soil profile. Most measures responded positively to experimental N addition. Effect sizes were generally greater for bulk soil than biocrust. Results were incorporated into a meta-analysis of arid ecosystem responses to N amendment that included data from 14 other studies. Effect sizes were calculated for biomass and metabolic responses. Regressions of effect sizes, calculated for biomass, and metabolic responses, showed similar trends in relation to N application rate and N load (rate × duration). The critical points separating positive from negative treatment effects were 88 kg ha^-1^ y^-1^ and 159 kg ha^-1^, respectively, for biomass, and 70 kg ha^-1^ y^-1^ and 114 kg ha^-1^, respectively, for metabolism. These critical values are comparable to those for microbial biomass, decomposition rates and respiration reported in broader meta-analyses of N amendment effects in mesic ecosystems. However, large effect sizes at low N addition rates indicate that arid ecosystems are sensitive to modest increments in anthropogenic N deposition.

## Introduction

Drylands (arid and semiarid lands) comprise about 35% of the terrestrial surface of the western US and 41% of global terrestrial lands (Pointing and Belnap, 2012). Over 35% of the world’s human population depends on dryland ecosystems for their livelihood, and this number is increasing (Millennium Ecosystem Assessment, 2003). These resource-limited ecosystems have low resilience and resistance to abiotic perturbations. Thus environmental changes often have large ecological effects on both regional (Mack and Thompson, 1982; MacMahon, 1987) and global scales (Ahlström et al., 2015). One perturbation of concern is the atmospheric deposition of nitrogen (N), which increases as human utilization of these lands expands. Therefore, it is important for management of dryland ecosystems to understand their vulnerability and response to N deposition.

Globally, the responses of soil microbial communities to experimental N manipulations have been extensively studied, and the subject of several meta-analyses (Knorr et al., 2005; Treseder, 2008; Janssens et al., 2010; Zhou et al., 2014). However, few studies of arid ecosystems had been conducted at the time of writing. There are multiple reasons why arid ecosystem responses to N might differ from those of wetter mesic biomes. Foremost, responses to nutrient amendments are contingent on the annual distribution of soil moisture, which controls plant production and soil processes (Stursova et al., 2006; Ladwig et al., 2012; Collins et al., 2014). In addition, desert soils differ from mesic soils in pH, organic matter concentration, and microbial community composition, both bacterial, and fungal (Porras-Alfaro et al., 2011; Fierer et al., 2012; Steven et al., 2013, 2014; Mueller et al., 2015). Proteolytic and phenol oxidase activities, which are both associated with N mineralization, are greater relative to glycosidase activities (Sinsabaugh et al., 2008; Hofmockel et al., 2010). And fungi are major agents of denitrification (Crenshaw et al., 2008; Chen et al., 2015).

Another contrast between deserts and mesic regions is the presence of biocrusts, a soil surface community of lichens, mosses, cyanobacteria, bacteria, and fungi. Because plant cover is low in deserts, biocrusts are often the dominant living cover (Belnap and Lange, 2003). In very hot deserts, the biomass of these surface crusts is low and dominated by heterotrophic bacteria and fungi. As soil moisture increases, cyanobacteria increase in abundance, followed by mosses and lichens. As rainfall increases, so does the importance of biocrusts in soil stability, the contribution of newly fixed C and N to soils, and the interception of nutrient-rich dust that includes anthropogenically created atmospheric N (Pointing and Belnap, 2012). The fungal networks that integrate biocrusts are able to translocate this N to plants (Green et al., 2008; Zhuang et al., 2014).

Given the low N content of arid soils and generally low rates of atmospheric N deposition at this site and US deserts in general (∼2 kg ha^-1^ y^-1^ in the absence of anthropogenic influence; Fenn et al., 2003), these ecosystems should be responsive to increases in N loading such as those associated with urbanization. The few experimental studies to date show varied responses (Supplementary Table S1). Interpretation is complicated by differences in N dose and duration of experiments, lack of redundancy in the response variables measured, and the spatial heterogeneity of arid landscapes.

We approached these issues two ways. First, we added N to a shrubland ecosystem that covers large areas of the southwestern U.S. Measurements included a broad range of nutrient, biomass and process responses nested within the major structural components of the landscape. Second, we assembled data from other N addition studies conducted in arid ecosystems for a meta-analysis that compared the sensitivity and responsiveness of arid ecosystems to those reported elswhere for mesic ecosystems. Both approaches indicate that arid soils are highly responsive to relatively small increments in N loading and that increased N availability accentuates differences among habitat patches.

## Materials and Methods

### Study Site

The study site is located in the Lake Mead National Recreation Area (Reno, NV, USA, UTM Zone 11 702869N 3927243E). Three randomly selected 100 m × 100 m sites, separated by at least 5 km, were chosen within *Larrea tridentata* (creosote bush) – *A. dumosa* (burro bush) dominated shrubland found on lower elevation alluvial fans within a 500 ha area designated as open for study by the U.S. National Park Service. The alluvial soils have an average texture of 80% sand, 13% silt, and 7% clay with a bulk pH of 7.4. Organic carbon content in the upper 10 cm averaged 0.35%. A nearby weather station has documented long-term average annual high and low temperatures of 23.8 and 11.1C, respectively, with an annual average precipitation of 19.8 cm. The nearest National Acid Deposition Program monitoring site (Red Rock Canyon National Conservation Area, 50 km west) reports wet N deposition rates of <1.0 kg/ha.

Within each site, fifteen *A. dumosa* plants of similar size, leaf area, and condition were selected. Each plant became the center of a 2 m × 2 m plot. To simulate N deposition typical of urban impacts, plots were fertilized with a nitrate-ammonium mix at three levels: 0, 7, and 15 kg N ha^-1^ yr^-1^, added in seven parts every 2 months from March 2012 to May 2013 to mitigate the effects of shock loading, sporadic precipitation and aeolian erosion. Plots were harvested in May 2013. Herein these treatments are referred to as Ambient (Amb), N7 and N15, respectively.

### Sampling

Pre-treatment soil and *Ambrosia* leaf samples were collected at each site from within the delineated plots. After the fertilization period, soil and plant leaf samples were again collected from the delineated plots. At both times, soils were collected to depths of 0–0.5 cm (hereafter referred to as “biocrust”) and 0–10 cm (hereafter referred to as “bulk soil”) from under each plant canopy (hereafter referred to as “canopy” samples) and from the open areas between plants (hereafter referred to as “interspace” samples). Within each plot, soil samples collected on the north, east, south, and west side of the plant at the stem, mid-canopy, and dripline were combined to form a single composite biocrust or bulk soil canopy sample. Interspace biocrust and soil samples were collected between the target *Ambrosia* and its nearest neighbor on the north, east, south, and west. These samples were collected 50–100 cm away from the canopy edge of the target plant and combined to form a single composite biocrust or soil sample for each plot.

Biocrust and bulk soil samples were divided into four parts for analysis of bacterial and fungal abundance, chlorophyll *a*, enzyme activities, and chemistry. All soils were immediately placed on ice until reaching the laboratory. Samples for molecular and enzymatic analyses were frozen at -70°C until analyzed.

### Chemical Analyses

Subsamples of the collected soils were air-dried, sieved to 2 mm and mixed; plant leaves were dried and ground, and both soil and plant materials then sent to the Plant and Soil Analysis Laboratory at Brigham Young University in Provo, UT, USA. Organic matter was determined by chromic acid digestion (Walkley and Black, 1934). Sample pH was measured in a saturated soil paste solution (Rhoades, 1982). Total N (TN) was determined by Kjeldahl analysis (Bremner and Keeney, 1966). Available phosphorus (P_av_) and available K (K_av_) were extracted with NaHCO_3_ (Olsen et al., 1954; Schoenau and Karamonos, 1993, respectively).

P_av_ was quantified colorimetrically at 880 nm using the vanadomolybdophorphoric assay (Rice et al., 2012). K_av_ was analyzed using an ICP spectrometer (Johnson and Ulrich, 1959). Soil particle size distributions were determined by the hydrometer method (Day, 1965). Plant leaves were dried, ground, digested with perchloric acid and analyzed for total N (Bremner and Keeney, 1966) and minerals, using ICP spectrometry (Johnson and Ulrich, 1959).

Net nitrification and ammonification rates were calculated by measuring NO_3_-N and NH_4_-N concentrations in KCl extracts before and after a 10 day incubation at 20C (Finzi et al., 2006). N Mineralization was calculated as the sum of nitrification and ammonification. Net changes were calculated in units of μg g^-1^ d^-1^.

### Chlorophyll

Chlorophyll *a* was extracted from biocrust in acetone and quantified by HPLC based on peak areas from a photodiode array detector at 436 nm, using commercial standards (DHI Water and Environment, Denmark; Karsten and Garcia-Pichel, 1996).

### Microbial Abundance

DNA was extracted from 0.5 g soil samples collected from Site 1 using the FAST DNA Spin kit for Soil following manufacturer recommendations (MP Biomedicals, Solon, OH, USA). Extracted DNA was quantified using the Quant-it PicoGreen dsDNA Assay Kit (Invitrogen, Carlsbad, CA, USA), measured on a BioTech Synergy H1 plate reader.

The DNA concentrations were normalized to 1 ng/μl, and quantitative PCR (qPCR) reactions were conducted in 96-well plates on a CFX Connect Real-Time PCR System (BioRad Laboratories, Hercules, CA, USA), using a modified procedure from Castro et al. (2010). DNA quantitative standards for bacterial and fungal rRNA genes were generated by amplifying the bacterial 16S RNA gene from *Microcoleus vaginatus* (the most common cyanobacterium in regional biocrusts) using EUB 338 and EUB 518 primers (Lane et al., 1985), or amplifying the fungal SSU RNA gene from a *Phoma* sp. culture (one of the most abundant fungi in regional soils) using the nu-SSU- 1196F and nu-SSU-1536R primers (Borneman and Hartin, 2000). Amplified fragments were cloned into *Escherichia coli* to generate a single copy bacterial or fungal gene clone for generation of standard curves. For field sample DNAs, duplicate 30 μl qPCR reaction contained 15 ul of iQ SYBR Green Supermix (BioRad Laboratories, Hercules, CA, USA), 1.25 μg/μl BSA (Roche Diagnostics GmbH, Mannheim Germany), 1 μl of normalized soil DNA and 133 nM of each primer (EUB 338 and EUB 518 Bacterial; Fungal Primers: nu-SSU- 1196F and nu-SSU-1536R137). The reactions were amplified using the following conditions: 95C for 3.25 min followed by 40 cycles of 95C for 15 s, annealing temperature for 30 s (55C bacterial or 53C fungal) and 72C for 30 s, followed by a step at 95C for 1 min, and 80 cycles at 55C for 10 s with a 4C hold for dissociation curve analysis.

### Soil Enzyme Activities

Biocrust and bulk soil samples were assayed for the potential activities of β-1,4-glucosidase (BG), alkaline phosphatase (AP), alanine aminopeptidase (AAP), and β-1,4-*N*-acetylglucosaminidase (NAG) using flourigenic methylumb- elliferyl-linked substrates, following the protocol of Stursova et al. (2006). Aliquots (200 μl) of sample suspensions (1 g sample homogenized in 125 ml of 50 mM bicarbonate buffer, pH 8) were dispensed into 96-well microplates. Each microplate included 16 replicate wells per assay, plus positive and negative controls for quench correction. Microplates were incubated at 21C. Fluorescence was measured at excitation and emission wavelengths of 365 and 450 nm, respectively, using a BioTech Synergy H1 plate reader. Activities were calculated in units of nmol g^-1^ h^-1^.

#### Data Analyses

Data were grouped into three categories for multivariate analysis: (i) soil nutrients and N transformation processes, (ii) soil enzyme activities, and (iii) plant chemistry. For soil nutrients and ecoenzyme activities (EEA), we first used permutational MANOVA that included the fixed effects of N treatment, soil collection depth, and collection location including all interaction terms as well as the random effects of site and plot. Plot was nested within site and N treatment. Following normalization of response variables to equalize the measurement scales, perMANOVA was conducted in Primer v. 6.1.10 using 99999 iterations, residuals calculated under a restricted model, and type III partial SS (Clarke and Gorley, 2009). We included *a priori* contrasts to compare the N treatments. For foliar chemistry, the perMANOVA included the fixed effect of N treatment and the random effects of site and plant (nested within site and N treatment).

When multivariate analyses showed significant treatment effects, we followed up with general linear mixed effects models for individual response variables, including the same fixed and random factors described above (restricted maximum likelihood, Proc MIXED, SAS v. 9.3, SAS Institute Inc., Cary, NC, USA). All variables were natural log-transformed to meet assumptions of normality of residuals and homogeneity of variances, with the exception of ammonification, which did not require transformation. Degrees of freedom of these models varied due to rare incidences of missing data, and the exclusion of one outlier (an ant nest) in the soil chemistry data. For each N treatment, we calculated an effect size as *RII* = (N treatment – Ambient)/(N treatment + Ambient) following Armas et al. (2004) to facilitate cross-response and cross-study comparisons.

The same individual general linear mixed models were used to analyze the responses of soil organic carbon (SOC): Total N and SOC:P_av_ ratios as well as microbial biomass indicators. These variables were log-transformed prior to analysis. Because soil fungi and bacteria data were collected only for Site 1, these models did not include the random effect of site. Chlorophyll data were collected only for the biocrust, so soil depth was not a factor in that analysis.

Mantel tests were conducted to examine relationships among soil nutrient, ecoenzyme, and foliar chemistry response matrices (RELATE function, 99999 permutations, Spearman *Rho*, Primer v. 6.1.10, Clarke and Gorley, 2009). First, across the entire dataset (180 observations) we examined the relationship between soil nutrients/processes and EEA. Second, for the matrices of responses observed at the scale of plants (45 observations), we examined relationships among foliar chemistry, soil nutrients/processes and EEA measured beneath the plant canopy (averaged over the 0.5 and 10 cm soil depths).

### Meta-Analysis of Published Studies

Because most studies of arid ecosystem responses to experimental N manipulation have only recently been published, these systems have not been well represented in previous meta-analyses. We created a comparative context for this study by assembling data from 14 studies (this study and 13 others) that included soil microbial responses to N amendment (Supplementary Table S1). N application rates ranged from 5 to 560 kg ha^-1^ y^-1^ with treatment durations of 0.3–10 year.

Eight studies (including our own) included microbial biomass responses, measured variously by phospholipid fatty acids (total, bacterial, fungal), qPCR (bacterial 16S gene copies, fungal 18S gene copies), biocrust chlorophyll, cyanobacteria species richness, muramic acid, and/or microbial biomass carbon or nitrogen (chloroform fumigation/extraction). For each N treatment, defined as a combination of application rate and treatment duration, we calculated the effect size *RII* as described above. For our Nevada study, we used the untransformed data to calculate LS means from the mixed effects model to use in RII. These effect sizes were pooled into a single category called microbial biomass responses.

Ten studies included microbial metabolic responses to N treatment measured using various enzyme assays (β-glucosidase, Ala/Leu-aminopeptidase, phosphatase, β-*N*-acetylglucosaminidase, urea aminohydrolase, invertase, phenol oxidase, peroxidase), BIOLOG substrate induced respiration (total change in absorbance), respiration (CO_2_ eﬄux), net ammonification and/or net nitrification. As above, we calculated an effect size for each N treatment and pooled the results into a single category called microbial metabolic responses.

Regression analysis was used to relate effect size (*RII*) to N application rate (kg ha^-1^ y^-1^) and cumulative N loading (application rate × duration of treatment). For studies that included multiple measures of biomass or metabolic responses per N treatment, only the mean effect size was used in the regression analyses so that all observations were independent. Analyses compared model fit between linear regression and log-transformation of the N treatment (*x*-axis) using relative *r*^2^ values. Within studies, the number of treatment replicates ranged from 2 to 10 (mean 5.4, Supplementary Table S1). However, we chose not to conduct weighted regressions because of the diversity of response metrics (i.e., weighting by study also biases some response variables over others).

## Results

### Soil Nutrient Levels and N Transformation Processes

**FIGURE 1:**
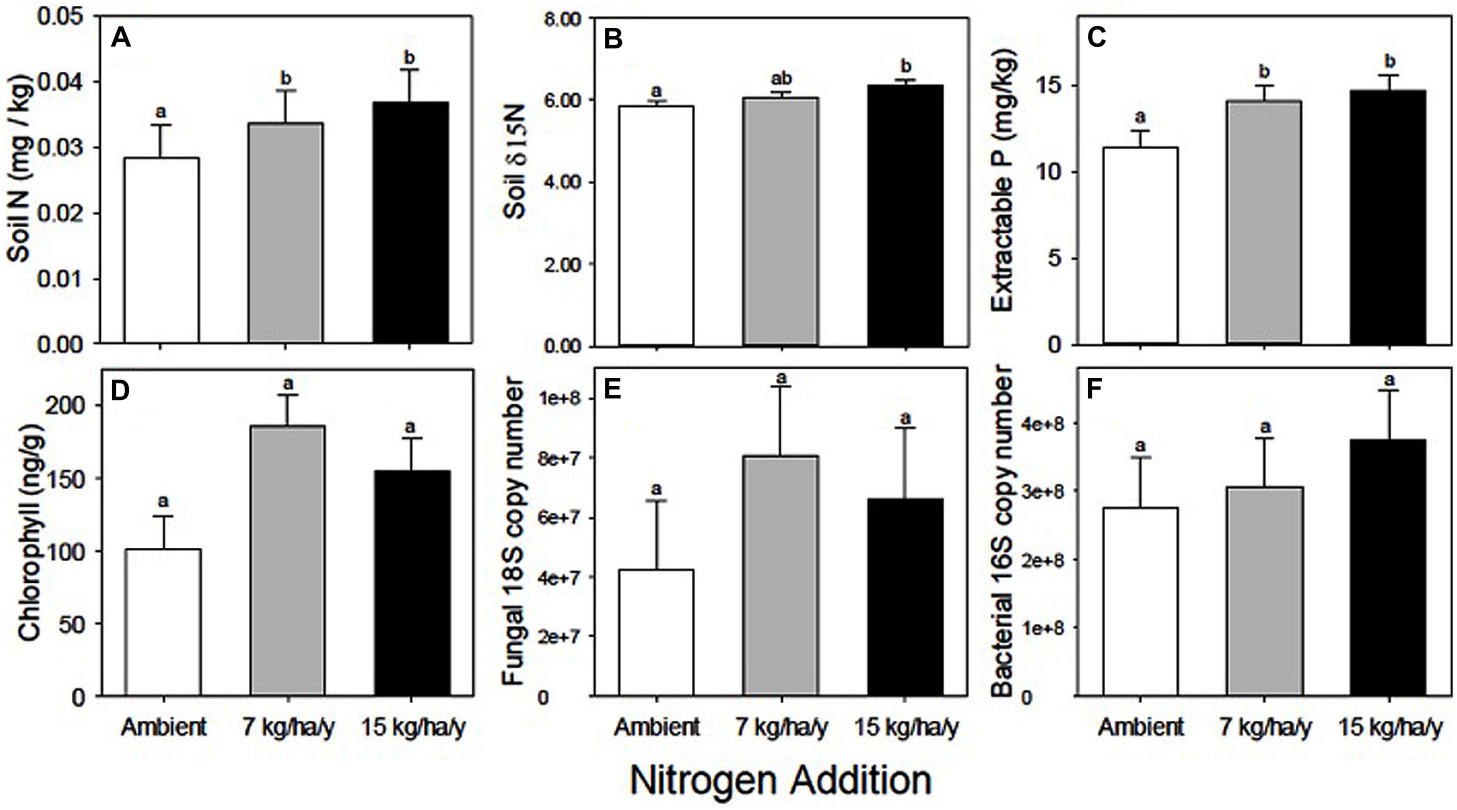
**Soil nutrient and microbial biomass responses to experimental N addition. (A)** Soil N. **(B)** Soil δ15N. **(C)** Extractable P. **(D)** Chlorophyll *a*. **(E)** Fungal 18S copy number/g. **(F)** Bacterial 16S copy number/g. Different letters indicate significant differences among the N treatments. Data summarized in **Table 1**. Statistical results presented in Supplementary Table S2.

Most soil nutrients and processes were influenced by N addition, with the exception of potassium (K), soil δ15N (**Figure 1B**), and N mineralization (Supplementary Table S2, perMANOVA *P* = 0.0003). Soil N increased by 15 and 30% in the N7 and N15 treatments relative to ambient plots, however, only the N15 response was significantly greater than ambient (**Table 1**; **Figure 1A**).

**Table 1:** Soil nutrient data by horizon, location, and N treatment.

Horizon	Location	N trt	Organic C		Total N		Available P		Available K		SOC:TN		SOC:Pav	
			%	SD	%	SD	ng/g	SD	ng/g	SD	Ratio	SD	Ratio	SD
Biocrust 0.5 cm	Canopy	Amb	0.521	0.271	0.045	0.021	14.07	3.61	257.9	93.0	12.1	3.4	915	360
Biocrust 0.5 cm	Interspace	Amb	0.269	0.121	0.021	0.010	9.87	2.05	115.7	45.8	15.4	8.8	692	281
Soil 10 cm	Canopy	Amb	0.362	0.300	0.029	0.009	10.95	2.95	329.6	98.3	11.4	2.1	887	816
Soil 10 cm	Interspace	Amb	0.128	0.060	0.014	0.004	10.64	3.53	127.1	35.1	11.0	3.7	343	198
Biocrust 0.5 cm	Canopy	N7	0.698	0.309	0.060	0.022	16.03	5.63	275.0	151.0	13.8	4.2	1181	522
Biocrust 0.5 cm	Interspace	N7	0.310	0.135	0.024	0.010	11.16	2.56	112.9	57.0	16.0	8.1	754	352
Soil 10 cm	Canopy	N7	0.297	0.164	0.034	0.016	14.93	6.31	336.9	111.7	9.9	1.8	507	122
Soil 10 cm	Interspace	N7	0.155	0.058	0.017	0.005	14.10	4.23	136.8	65.7	10.8	2.2	306	141
Biocrust 0.5 cm	Canopy	N15	0.594	0.231	0.061	0.025	17.53	6.20	257.5	78.2	12.5	4.9	901	278
Biocrust 0.5 cm	Interspace	N15	0.265	0.161	0.030	0.015	12.78	3.81	127.2	58.7	10.4	3.3	522	220
Soil 10 cm	Canopy	N15	0.329	0.112	0.038	0.011	15.30	4.77	417.7	139.8	10.5	2.7	565	149
Soil 10 cm	Interspace	N15	0.136	0.067	0.018	0.007	12.97	3.79	164.9	29.6	9.2	4.1	292	151

*SOC, soil organic carbon; TN, total N; Pav, available P.*

**FIGURE 2:**
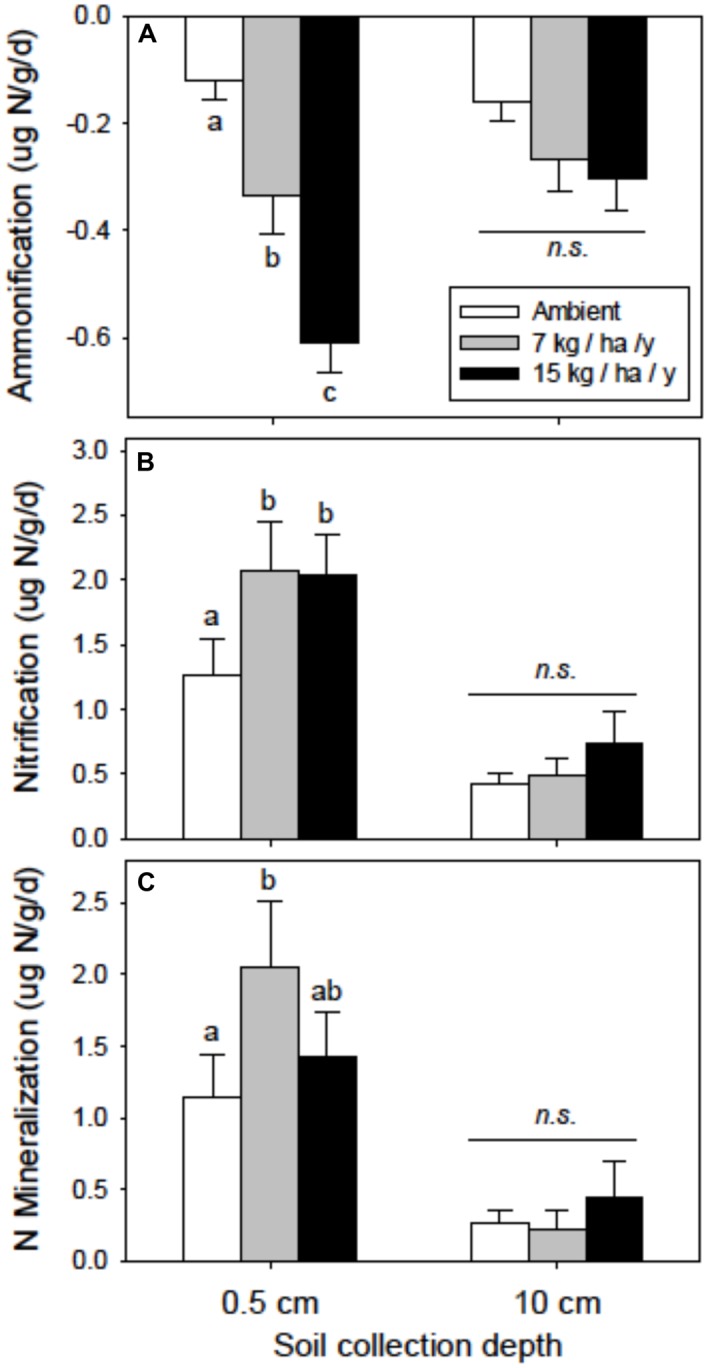
**N transformation rates in relation to experimental N addition for biocrust (0.5 cm) and soil (0–10 cm). (A)** Ammonification. **(B)** Nitrification. **(C)** N mineralization. Different letters indicate significant differences among the N treatments. Data summarized in **Table 2**. Statistical results presented in Supplementary Table S2.

The effects of N addition varied more strongly with soil depth than with spatial location (Supplementary Table S2: perMANOVA N × Depth *P* = 0.027; N × Location *P* = 0.735). This distinction was most pronounced for net ammonification, which declined from -0.12 (Amb) to -0.33 (N7) to -0.61 (N15) μg N g^-1^ d^-1^ for the biocrust horizon and from -0.16 to -0.27 to -0.30 μg N g^-1^ d^-1^, respectively, in the bulk soil (**Table 2**; **Figure 2A**, full statistical results in Supplementary Table S2). Net nitrification also responded significantly to N addition but only in the biocrusts (increasing from 1.27 to 2.12 to 2.04 μg N g^-1^ d^-1^, respectively, **Table 2**; Supplementary Table S2; **Figure 2B**), although the Nitrification × Depth interaction was marginally non-significant (*P* = 0.098). Net N mineralization in the biocrust followed a similar pattern, responding more strongly in the N7 treatment than the N15 treatment (from 1.15 to 2.05 to 1.43 μg N g^-1^ d^-1^, respectively), but again treatment effects were non-significant over the deeper soil profile (**Table 2**; **Figure 2C**).

**Table 2:** N transformation rates by horizon, location, and N treatment.

Horizon	Location	N trt	Nitrification		Ammonification		N mineralization	
			μg g^-1^ d^-1^	SD	μg g^-1^ d^-1^	SD	μg g^-1^ d^-1^	SD
Biocrust 0.5 cm	Canopy	Amb	2.299	1.700	-0.098	0.289	2.200	1.902
Biocrust 0.5 cm	Interspace	Amb	0.348	0.311	-0.124	0.055	0.224	0.295
Soil 10 cm	Canopy	Amb	0.712	0.575	-0.239	0.236	0.473	0.604
Soil 10 cm	Interspace	Amb	0.113	0.095	-0.095	0.115	0.018	0.141
Biocrust 0.5 cm	Canopy	N7	4.275	2.361	-0.251	0.758	4.025	2.626
Biocrust 0.5 cm	Interspace	N7	0.634	0.320	-0.272	0.132	0.362	0.348
Soil 10 cm	Canopy	N7	0.829	0.801	-0.404	0.436	0.424	1.004
Soil 10 cm	Interspace	N7	0.132	0.144	-0.151	0.122	-0.018	0.195
Biocrust 0.5 cm	Canopy	N15	3.115	1.629	-0.694	0.376	2.421	1.698
Biocrust 0.5 cm	Interspace	N15	0.832	0.569	-0.524	0.175	0.308	0.468
Soil 10 cm	Canopy	N15	1.323	1.694	-0.341	0.377	0.982	1.821
Soil 10 cm	Interspace	N15	0.191	1.252	-0.277	0.273	-0.468	1.373

Available P (**Table 1**) increased ∼25% with N addition (*RII* = 0.12, **Figure 1C**; Supplementary Table S1), from 11.4 mg/kg to 14.1 and 14.6 mg/kg in the N7 and N15 treatments, possibly as a result of increased acidity generated from nitrification, even though the N treatment did not significantly reduce bulk soil pH, which ranged from 7.1 to 7.4.

N addition also altered nutrient ratios (**Table 1**). For biocrust, the molar SOC:P_av_ ratio was 26% lower under the highest level of N addition relative to N7 and 11% lower relative to the control (N × Depth, *F*_2,124_ = 3.12, *P* = 0.0475, SOC:P_av_: Amb = 804, N7 = 967, N15 = 711). More generally, the SOC:P_av_ ratio decreased from 709 to 687 to 570 for the Amb, N7 and N15 treatments, but the trend was not statistically significant. The molar SOC:TN ratio showed a marginally non-significant decline with N addition (*F*_2,40_ = 2.84, *P* = 0.0705, C:P: Amb = 12.6 ± 0.8, N7 = 12.5 ± 0.8, N15 = 10.7 ± 0.8).

Other nutrient variables were non-responsive to N addition, but showed significant spatial patterns. SOC was significantly greater beneath the shrub canopy (mean ± SE: 0.47 ± 0.04%) than in the interspace (0.21 ± 0.04%; Supplementary Table S2, *P* < 0.0001) and declined 89% with soil depth from 0.44 ± 0.04% at 0.5 cm to 0.23± 0.04% at 10 cm (**Table 1**; Supplementary Table S2). Soil N was 63% greater in the biocrust (0.041 ± 0.005) than in the bulk soil (0.025 ± 0.005), and 118% greater beneath the shrub canopy (0.045 ± 0.005) than in interspaces (0.021 ± 0.005, **Table 1**; Supplementary Table S2). Available P was 24% greater under the shrub canopy (14.82 ± 0.78) than in interspaces (11.92 ± 0.78, **Table 1**; Supplementary Table S2). Soil K was also greater (139%) beneath the shrub canopy (312 ± 19) compared to the interspaces (131 ± 19, **Table 1**; Supplementary Table S2). Biocrust soils beneath shrub canopies had 400% greater net nitrification rates (3.02 ± 0.16) than interspace biocrust (0.60 ± 0.16); this difference was stronger (620%) for bulk soils (canopy 0.96 ± 0.16; interspace 0.13 ± 0.16; **Table 2**; Supplementary Table S2, *P* = 0.0002). Net N mineralization showed a similar trend (**Table 2**). Rates for biocrust beneath shrub canopy were 10-fold greater than those for interspace crust (2.88 vs. 0.30), with a stronger pattern across the deeper soil profile (canopy: 0.63, interspace: -0.16).

### Fungal, Bacterial, and Cyanobacterial Biomass

None of the estimates of microbial biomass responded significantly to N amendment in either soil horizon, and there was little shift in fungal/bacterial ratios (∼0.15, Supplementary Table S4). However, in nearly all cases, the biomass indicators increased with N addition (**Table 3**; **Figures 1D–F**). Sample sizes for determining treatment effects on fungal and bacteria copy numbers were small (*n* = 5 plots) because measurements were conducted only at Site 1. Fungal copy numbers were 90% greater for the N7 treatment (*RII* = 0.31) and 56% greater for N15 (*RII* = 0.22) relative to the Amb plots (**Figure 1E**). Bacterial copy numbers were 11% greater for the N7 treatment (*RII* = 0.05) and 36% higher for N15 (*RII* = 0.15; **Table 3**; **Figure 1F**). Approximately 3% of the bacterial sequences were cyanobacteria, which is typical for hot, dry deserts (Fierer et al., 2012). Biocrust chlorophyll *a*, which was measured at all three sites, was 82% greater for the N7 treatment (*RII* = 0.29) and 52% greater for N15 (*RII* = 0.21) relative to the Amb plots (**Table 4**; **Figure 1D**).

**Table 3:** Fungal and bacterial SSU rRNA gene copy number by horizon, location, and N treatment.

Horizon	Location	N trt	Fungal 18S		Bacterial 16S		Fungi/Bacteria	
			Copies/g	SD	Copies/g	SD	Ratio	SD
Biocrust 0.5 cm	Canopy	Amb	1.03E+08	6.20E+07	5.82E+08	2.47E+08	0.169	0.056
Biocrust 0.5 cm	Interspace	Amb	2.48E+07	1.27E+07	1.88E+08	2.74E+07	0.140	0.091
Soil 10 cm	Canopy	Amb	3.42E+07	1.12E+07	2.35E+08	4.64E+07	0.146	0.039
Soil 10 cm	Interspace	Amb	7.08E+06	3.65E+06	1.04E+08	2.98E+07	0.066	0.018
Biocrust 0.5 cm	Canopy	N7	2.28E+08	2.17E+08	6.22E+08	2.87E+08	0.345	0.217
Biocrust 0.5 cm	Interspace	N7	1.94E+07	6.66E+06	2.42E+08	1.02E+08	0.089	0.032
Soil 10 cm	Canopy	N7	6.50E+07	4.67E+07	2.34E+08	1.01E+08	0.366	0.440
Soil 10 cm	Interspace	N7	9.14E+06	2.44E+06	1.29E+08	3.51 E+07	0.072	0.012
Biocrust 0.5 cm	Canopy	N15	1.42E+08	1.17E+08	7.18E+08	3.97E+08	0.204	0.105
Biocrust 0.5 cm	Interspace	N15	6.24E+07	9.93E+07	2.87E+08	1.06E+08	0.221	0.335
Soil 10 cm	Canopy	N15	5.82E+07	5.92E+07	3.97E+08	2.44E+08	0.148	0.097
Soil 10 cm	Interspace	N15	7.40E+06	2.10E+06	1.09E+08	8.78E+07	0.094	0.059

**Table 4:** Biocrust chlorophyll *a* data by horizon, location, and N treatment.

Horizon Location	N trt	Chlor *a*	
		ng g^1^	SD
Biocrust 0.5 cm Canopy	Amb	135.0	86.4
Biocrust 0.5 cm Interspace	Amb	68.4	36.8
Biocrust 0.5 cm Canopy	N7	234.2	136.2
Biocrust 0.5 cm Interspace	N7	75.5	55.1
Biocrust 0.5 cm Canopy	N15	265.8	214.9
Biocrust 0.5 cm Interspace	N15	104.4	83.5

Independent of responses to N amendment, microbial biomass was greater in the biocrust than in the bulk soil (217% for fungi and 118% for bacteria). Biocrust chlorophyll was 156% higher under the canopy (211.7 ± 17.9) than in interspaces (82.8 ± 17.9).

### Soil Enzyme Activity

**FIGURE 3:**
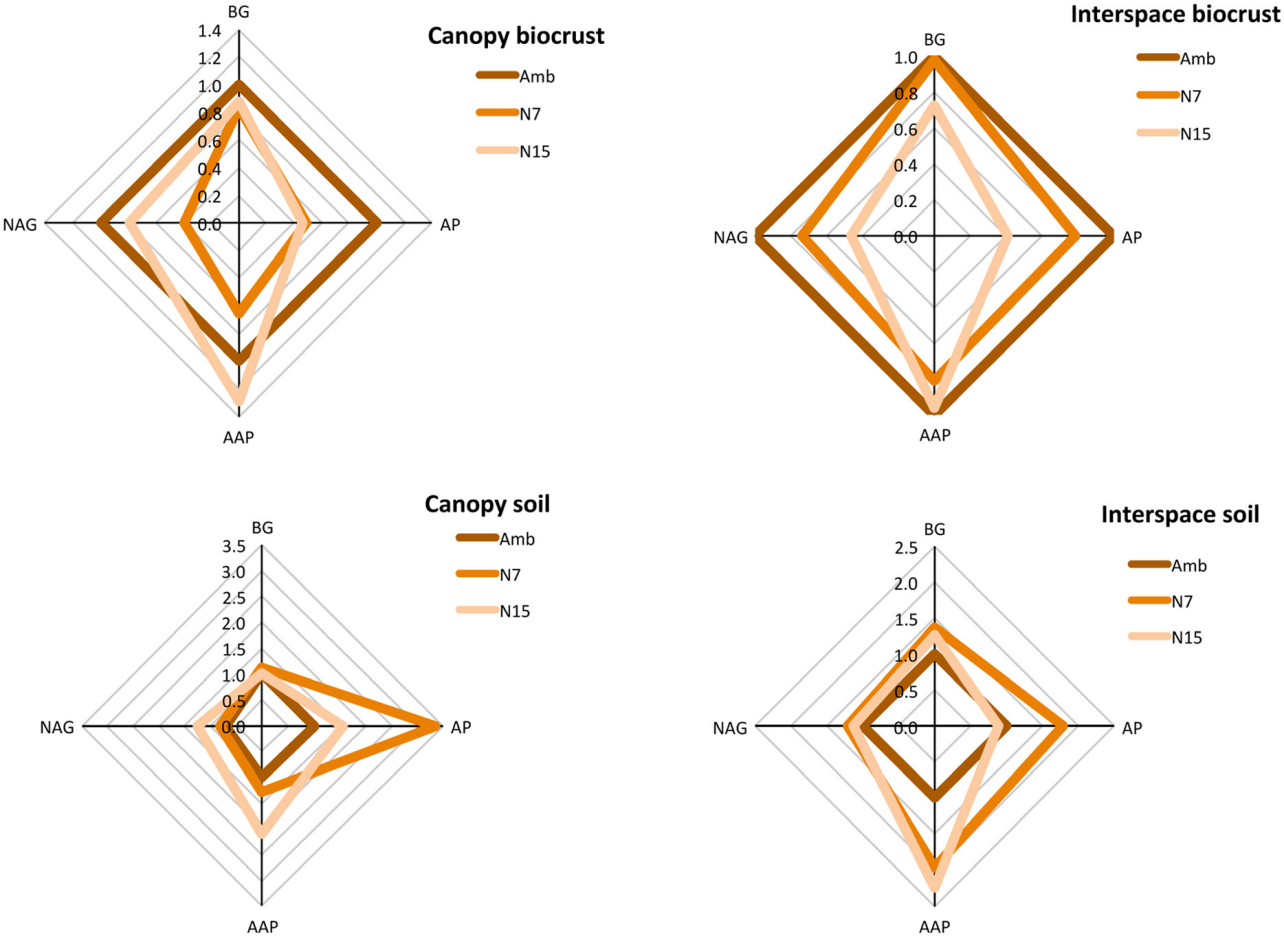
**Relative responses of soil ecoenzyme activity to N treatment for biocrusts (0.5 cm) and bulk soil (0–10 cm).** BG: β-1,4-glucosidase, AP: alkaline phosphatase, AAP: alanine aminopeptidase, NAG: β-1,4-*N*-acetylglucosaminidase.

Collectively, the four soil EEA responded significantly to N addition (**Table 5**; Supplementary Table S3, **Figure 3**: perMANOVA *P* = 0.0039). AAP was the most responsive, increasing with N addition in all four soil fractions. Overall, AAP activity increased by 54% (*RII* = 0.21, Amb = 21.64 ± 5.02, N7 = 22.83 ± 5.02, N15 = 33.33 ± 5.02; Supplementary Table S3). In contrast, β-1,4-*N*-acetylglucosaminidase activity declined 36% across treatments (*RII* = -0.22; Amb = 5.25 ± 0.96, N7 = 3.87 ± 0.96, N15 = 3.37 ± 0.96; Supplementary Table S3).

**Table 5:** Ecoenzymatic activity by horizon, location, and N treatment.

Horizon	Location	N trt	AAP		AP		NAG		BG		AG	
			nmol g^-1^ d^-1^	SD	nmol g^-1^ d^-1^	SD	nmol g^-1^ d^-1^	SD	nmol g^-1^ d^-1^	SD	nmol g^-1^ d^-1^	SD
Biocrust 0.5 cm	Canopy	Amb	43.0	29.7	38.9	29.2	15.3	14.8	56.7	32.2	2.91	2.89
Biocrust 0.5 cm	Interspace	Amb	31.4	26.5	32.8	26.5	2.94	2.06	26.6	31.2	2.48	7.14
Soil 10 cm	Canopy	Amb	16.0	11.8	6.34	7.08	2.28	1.87	18.5	9.9	1.28	2.64
Soil 10 cm	Interspace	Amb	19.0	14.9	4.43	4.86	0.71	0.55	11.7	12.3	0.31	0.36
Biocrust 0.5 cm	Canopy	N7	34.8	22.7	30.0	32.2	11.4	16.0	55.4	35.5	4.13	5.68
Biocrust 0.5 cm	Interspace	N7	17.6	11.2	16.2	16.8	1.38	1.49	22.4	21.7	0.57	0.58
Soil 10 cm	Canopy	N7	29.7	25.4	15.3	18.2	2.77	2.53	25.1	16.8	1.00	1.53
Soil 10 cm	Interspace	N7	22.4	18.8	17.9	18.3	0.54	0.54	12.4	14.8	0.29	0.42
Biocrust 0.5 cm	Canopy	N15	41.2	23.3	14.8	13.7	7.20	4.69	40.9	23.2	3.31	3.95
Biocrust 0.5 cm	Interspace	N15	33.7	18.1	14.7	15.9	2.79	3.30	23.2	15.0	1.37	1.31
Soil 10 cm	Canopy	N15	32.9	22.2	7.35	11.3	2.58	1.92	23.2	14.7	1.71	1.94
Soil 10 cm	Interspace	N15	34.5	30.5	7.31	4.21	0.90	0.91	11.2	9.3	1.19	1.70

*AAP, alanine aminopeptidase; AP, alkaline phosphatase; NAG, β-*N*-acetylglucosaminidase; BG, β-glucosidase; AG, α-glucosidase.*

Like N transformation rates, the effects of N addition on ecoenzyme activity varied with sampling depth (**Table 5**; Supplementary Table S3, **Figure 3**; perMANOVA *P* = 0.0045). This interaction was driven primarily by AP, which declined 54% with N addition in biocrust soil (*RII* = -0.37; Amb = 29.98 ± 8.89, N7 = 21.01 ± 8.89, N15 = 13.76 ± 8.89) but increased 59% for bulk soil (*RII* = 0.23; Amb = 4.16 ± 8.89, N7 = 13.70 ± 8.89, N15 = 6.60 ± 8.89; **Figure 3**). β-1,4-*N*-acetylglucosaminidase showed a similar pattern with a 45% decline with N in the biocrust (*RII* = -0.29; Amb = 9.00 ± 1.28, N7 = 6.37 ± 1.28, N15 = 4.99 ± 1.28) but a 17% increase in bulk soil (*RII* = 0.08; Amb = 1.49 ± 1.28, N7 = 1.36 ± 1.28, N15 = 1.74 ± 1.28). AAP also showed a greater relative response to N in deep soil (116%, *RII* = 0.37) compared to surface biocrust (23%, *RII* = 0.10).

Spatially, all ecoenzymes showed greater potential activity in canopy soils than in interspaces (**Table 5**). β-1,4-*N*-acetylglucosaminidase showed the strongest difference (353% greater in canopy soil) followed by β-glucosidase (107%), AAP (39%), and AP (37%).

### Foliar Nutrients

N addition had no effect on nutrient concentrations in leaves of *A. dumosa* (Supplementary Table S5, perMANOVA pseudo-*F* = 0.90, *P* = 0.4149).

### Connecting Biomass, Activities, and Nutrients

At the plot scale, the enzyme response matrix was positively associated with the nutrient response matrix, but only beneath the shrub canopy (Mantel ρ = 0.152, *P* = 0.0313, *n* = 45 plots), not within the interspaces (Mantel ρ = 0.088, *P* = 0.1849). Across all samples, the ecoenzyme response matrix was positively correlated with all estimates of microbial biomass. Fungal copy number showed the strongest positive correlation with ecoenzyme NMS axis1 (Spearman *r* = 0.39, *P* = 0.0026, *n* = 58), followed closely by bacterial copy number (Spearman *r* = 0.38, *P* = 0.0036, *n* = 58), then chlorophyll *a* (Spearman *r* = 0.37, *P* = 0.0004, *n* = 87).

### Meta-Analysis

**FIGURE 4:**
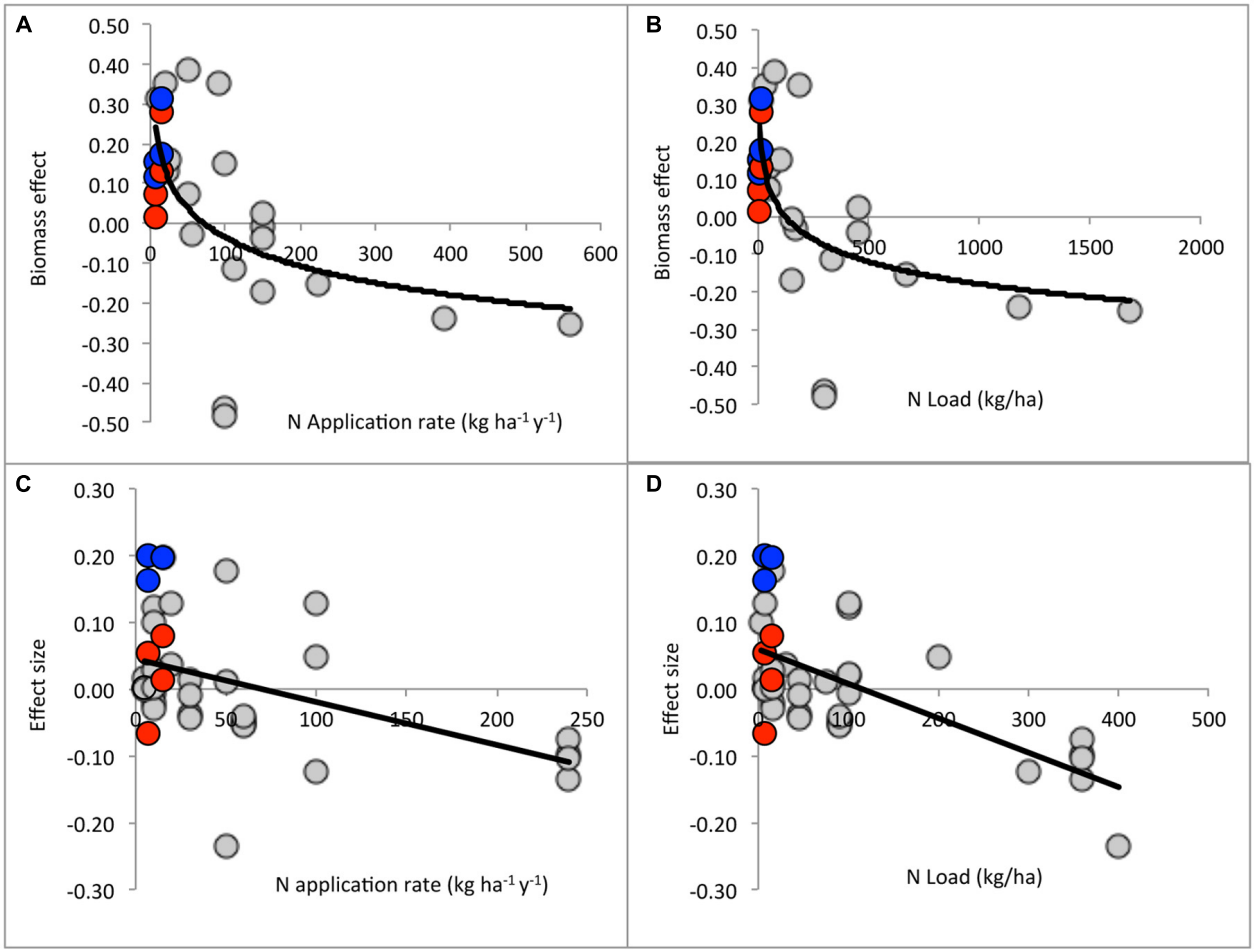
**Meta-analysis of aridland microbial responses to N addition. (A)** Microbial biomass in relation to N application rate: effect = -0.0959 (ln rate) + 0.429 (*r*^2^ = 0.358, *n* = 26, *p* = 0.00125). **(B)** Microbial biomass in relation to cumulative N load: effect = -0.0779 (ln load) + 0.395 (*r*^2^ = 0.386, *n* = 26, *p* = 0.0007). **(C)** Metabolic rates in relation to N application rate: effect = -0.00065 (rate) + 0.455 (*r*^2^ = 0.21, *n* = 41, *p* = 0.0024). **(D)** Metabolic rates in relation to cumulative N load: effect = -0.00052 (load) + 0.0593 (*r*^2^ = 0.41, *n* = 41, *p* < 0.00001). Blue circles represent Lake Mead bulk soil responses. Red circles represent Lake Mead biocrust responses. Data from Supplementary Table S1.

Across studies, microbial biomass responses to N treatment were best described by logarithmic regressions (**Figure 4**). For N application rate (kg ha^-1^ y^-1^): effect = -0.0959 (ln rate) + 0.429 (*r*^2^ = 0.358, *n* = 26, *p* = 0.00125). For N load: effect = -0.0779(ln load) + 0.395 (*r*^2^ = 0.386, *n* = 26, *p* = 0.0007). The critical points separating positive from negative treatment effects were 88 kg ha^-1^ y^-1^ and 159 kg ha^-1^, respectively.

Metabolic responses to N treatment were better described by linear regressions, rather than logarithmic (**Figure 4**). For application rate (kg ha^-1^ y^-1^): effect = -0.00065(rate) + 0.455 (*r*^2^ = 0.21, *n* = 41, *p* = 0.0024). For load: effect = -0.00052(load) + 0.0593 (*r*^2^ = 0.41, *n* = 41, *p* < 0.00001). The critical points separating positive from negative treatment effects were 70 kg ha^-1^ y^-1^ and 114 kg ha^-1^, respectively.

## Discussion

### N Responses and Spatial Heterogeneity

By most measures, nutrient availability, microbial biomass, and process rates were greater in soils beneath the shrub canopy compared to the interspace between plants, and greater in the surface biocrust horizon compared to the bulk soil. Most of these measures also responded positively to experimental N addition, despite the absence of large precipitation events over the study period or evidence of changes in plant production and nutrient content.

One notable exception was AP activity. Generally, phosphatase activity increases in response to experimental N addition because mitigating N limitation increases the relative demand for P (Skujiņš, 1978; Sinsabaugh and Follstad Shah, 2012). In that context, the 50% loss of phosphatase activity in the N-amended biocrusts is anomalous, particularly because activity increased by 50% over the deeper 10 cm profile (**Figure 3**).

Chemical analysis showed that available P increased by 20–50% with N addition in all soil fractions (**Table 1**). These P increases could be the result of acidity generated by increased nitrification, which increased significantly (60–80%) in biocrusts, with a somewhat smaller response for bulk soils. Analysis of these carbonate soils conducted prior to treatment found a total P content of 950 ± 40 μg/g (data not presented), which compared to values of 10–18 μg/g for P_av_ (**Table 1**) suggests a potential for increased P solubilization in response to acidity. Increased P_av_ may also be a product of greater microbial biomass in the N addition treatments. In any case, the divergent phosphatase responses of biocrust and bulk soil indicate that N addition depressed relative P limitation within the biocrust, but increased it for bulk soil, possibly as a result of nutrient competition with plants.

Although phosphatase is the clearest case of divergent responses between biocrust and bulk soil, the general trend extends to other ecoenzymes. Activities tend to show larger and more positive responses in the bulk soil than in the biocrust (**Figures 3** and **4**). This spatial pattern is consistent with the dose-dependent responses observed across studies (**Figure 4**). The surface biocrust likely experienced a greater effective N dose than the underlying mineral soil given the sparse precipitation over the study period. In addition, the mineral soil is the site of plant–microbe interaction, including carbon priming and nutrient competition, which may also affect the responses to N addition.

Only one other aridland study included measurements of the same ecoenzyme responses presented here (Stursova et al., 2006). That study, conducted in semiarid grassland, sampled soil (0–5 cm) beneath bunch grass (grama) canopy and in biocrust-covered interspaces. β-*N*-acetylglucosaminidase and β-glucosidase activities were similar in magnitude to those reported here, but AAP and AP activities were 3–5 times greater in the prior study. In response to N amendment (10 kg ha^-1^ y^-1^), AAP activity declined rather than increased, β-*N*-acetylglucosaminidase activity doubled, while AP and β-glucosidase activities showed little response.

### Integrating N Responses through Carbon Use Efficiency

The comparison above highlights the need for integrative measures of ecosystem response to N loading, rather than focusing on the causal nexus underlying individual responses. Across the patch mosaic landscape of arid shrubland, nutrient concentrations, microbial activity, microbial biomass and their responses to N addition are often correlated because they are integrated through microbial carbon use efficiency (CUE). CUE is commonly defined as the ratio of microbial growth to assimilation, which is often estimated as the sum of growth and respiration. But CUE can also be estimated from stoichiometric relationships among substrate composition, biomass composition and nutrient acquisition activities (Sinsabaugh et al., 2013). Sinsabaugh and Follstad Shah (2012) proposed that:

$$\mathrm{CUE}\;=\;{\mathrm{CUE}}_{\max}[S_{C:N}/(S_{C:N}\;+\;K_{N})],\mathrm{where}$$

$$S_{C:N}\;=\;({1/EEA}_{C:N})(B_{C:N}{/L}_{C:N}).$$

Ecoenzyme activities_C:N_ is the ratio of C to N-acquiring ecoenzymatic activities, measured here as the ratio of BG/(NAG + AAP); B_C:N_ is the elemental C:N ratio of microbial biomass; L_C:N_ is the elemental C:N ratio of labile organic matter; K_N_ is a half saturation constant (0.5); and CUE_max_ is the upper limit for microbial growth efficiency (0.6).

**FIGURE 5:**
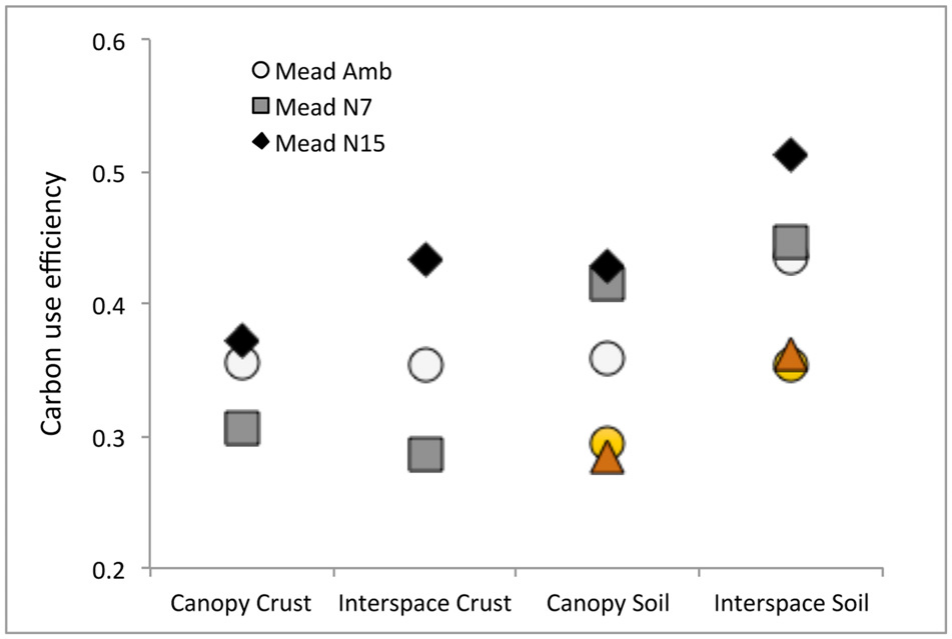
**Microbial carbon use efficiency (CUE) by horizon, location, and N treatment.** For comparison, the orange circles (Ambient) and triangles (N addition, 10 kg ha^-1^ y^-1^) are values calculated for a N addition study conducted in semiarid grassland in New Mexico (Stursova et al., 2006).

Using data from **Tables 1** and **5**, and assuming a mean value of 8.6 for B_C:N_ (Cleveland and Liptzin, 2007), the estimated CUEs ranged from 0.29 to 0.51 across soil fractions (**Figure 5**). For the N15 treatment, CUE increased in all soil fractions relative to the Amb. The N7 responses were mixed, CUE declined in biocrusts and increased in bulk soils. The CUE estimates for the semiarid grassland study conducted by Stursova et al. (2006) are similar to those for the shrubland (0.29–0.36), but show no net response to N addition after 10 years of fertilization (**Figure 5**). The Lake Mead NRA responses were measured after 1 year of treatment. Based on other cross study comparisons (**Figure 4**), this difference in CUE response between the studies may be the result of the 10-fold difference in cumulative N loading.

### Meta-Analysis of Aridland N Addition Studies

To aid decision making regarding ecosystem management and restoration, there has been an effort to define critical loads for N deposition by ecosystem type, where critical load is defined as “the deposition of a pollutant below which no detrimental ecological effect occurs over the long term according to present knowledge” (Pardo et al., 2011). This definition does not differentiate positive and negative responses. Our meta-analysis of aridland studies showed that N amendment had generally positive effects on microbial biomass and metabolic rates at N doses <70 kg ha^-1^ y^-1^ and N loads <120 kg ha^-1^, and generally negative effects at greater doses and loads (**Figure 4**).

Treseder (2008) conducted a meta-analysis of microbial biomass responses to N amendment, using data from 82 ecosystems (80 mesic and 2 arid). She found no significant differences across biomes, fertilizer types, or methods of biomass measurement. A regression relating ln(response ratio) to N load (*n* = 40, *r*^2^ = 0.18, *p* = 0.005) estimated the critical load separating net positive and net negative effects at 200 kg ha^-1^. Our regression model for aridland ecosystems yielded a similar critical load of 160 kg ha^-1^ (**Figure 3**).

The meta-analysis by Knorr et al. (2005) focused on litter decomposition rates, using data from 24 studies conducted in grassland, forest and tundra ecosystems. Litter decomposition was inhibited by N additions when dose rates were 2–20 times the ambient N deposition level or when litter quality was low (i.e., high lignin or humus content). Conversely, low ambient N deposition rates, high litter quality and short experiment duration tended to increase decomposition rates. The conflation of N dose with litter quality effects makes it difficult to estimate a critical value for the positive to negative transition, but dose rates >125 kg ha^-1^ y^-1^ generally inhibited decomposition. For comparison, our regression model for microbial metabolic responses in arid ecosystems estimates the critical dose at 70 kg ha^-1^ y^-1^.

Janssens et al. (2010) conducted a meta-analysis of N effects on heterotrophic respiration using data from 36 microcosm studies of temperate forest soils. On average, respiration declined by 15% in response to N amendment with a response range of -57 to +63%. Doses >50 kg ha^-1^ y^-1^ generally had negative effects, in comparison with our threshold estimate of 70 kg ha^-1^ y^-1^.

A meta-analysis by Zhou et al. (2014) analyzed N effects on soil respiration, resolving autotrophic and heterotrophic responses, using data from 295 studies conducted across a broad range of biomes. On average, N addition stimulated autotrophic respiration by 22% but reduced heterotrophic respiration by 13%. A regression relating the response ratio of heterotrophic respiration to N load showed no positive responses, i.e., the response ratio intercept was <1.0. The maximum N dose rate included in the meta-analysis was 74 kg ha^-1^ y^-1^, a value that approximates our estimated critical point for the transition from net positive to net negative effects.

Across ecosystems, lichens, and bryophytes, which are components of biocrusts, are among the most sensitive responders to increased N deposition with critical loads estimated at 1–9 kg ha^-1^ y^-1^; critical loads for semiarid grasses and shrubs also fall in this range (Pardo et al., 2011). Within our meta-analysis of arid soil responses, the lowest N application rate was 5 kg ha^-1^ y^-1^ and nearly all microbial responses <15 kg ha^-1^ y^-1^ were positive. These results suggest that arid ecosystems, characterized by low soil N contents and low atmospheric deposition rates, may be among the most sensitive to anthropogenic N deposition.

## Conclusion

Despite the difficulties in making direct comparisons among ecosystems, it appears that soil microbial responses to N amendment in arid ecosystems are broadly comparable to those of mesic ecosystems in terms of N saturation. However, it appears they are more sensitive to low dose inputs by some measures and more spatially heterogeneous in their responses. This heightened vulnerability will create many future challenges for land managers, as anthropogenically related N deposition is expected to increase as arid regions become more developed as spaces for living, recreation, and energy development.

## Conflict of Interest Statement

The authors declare that the research was conducted in the absence of any commercial or financial relationships that could be construed as a potential conflict of interest.
