# Supplementary material for: Soil microbial responses to nitrogen addition in arid ecosystems
Source: Front Microbiol. 2015 Aug 14;6:819. doi: 10.3389/fmicb.2015.00819 (PMC4536368; doi:10.3389/fmicb.2015.00819)
Supplement: Supplementary file 1 [file Data_Sheet_1.DOCX]

**SUPPLEMENTAL INFORMATION**

Table S1. Data sources for meta-analysis of aridland N amendment studies. Treatment responses are presented as effect sizes measured as RII = (N treatment – Control)/(N treatment + Control).

Table S2. Statistical results for soil nutrients and transformation processes by horizon, location and N treatment, including multivariate analysis (perMANOVA) that included all listed variables. Bold values indicate P < 0.05. All models included the random effects of site (three levels) and plant (nested within site and N treatment).

Table S3. Statistical results for ecoenzyme activities by horizon, location and N treatment, including multivariate analysis (perMANOVA) that included all listed variables. Bold values indicate P < 0.05. All models included the random effects of site (three levels) and plant (nested within site and N treatment).

Table S4. Statistical results for organismal responses by horizon, location and N treatment. Bold values indicate P < 0.05. All models included the random effects of site (three levels) and plant (nested within site and N treatment).

Table S5. Foliar chemistry of *Ambrosia dumosa*. There were no significant differences among N treatments.
